# Stability and sensitivity of water *T*_2_ obtained with IDEAL‐CPMG in healthy and fat‐infiltrated skeletal muscle

**DOI:** 10.1002/nbm.3654

**Published:** 2016-11-03

**Authors:** Christopher D.J. Sinclair, Jasper M. Morrow, Robert L. Janiczek, Matthew R.B. Evans, Elham Rawah, Sachit Shah, Michael G. Hanna, Mary M. Reilly, Tarek A. Yousry, John S. Thornton

**Affiliations:** ^1^ UCL Institute of Neurology, MRC Centre for Neuromuscular DiseasesLondonWC1N 3BGUK; ^2^ UCL Institute of Neurology, Neuroradiological Academic UnitLondonWC1N 3BGUK; ^3^ GlaxoSmithKlineUxbridgeUK

**Keywords:** Relaxometry, Water fat imaging, Muscle, IDEAL‐CPMG, HypoPP, Magnetic resonance imaging, Neuromuscular diseases, Musculoskeletal

## Abstract

Quantifying muscle water *T*_2_ (*T*_2_‐water) independently of intramuscular fat content is essential in establishing *T*_2_‐water as an outcome measure for imminent new therapy trials in neuromuscular diseases. IDEAL‐CPMG combines chemical shift fat–water separation with *T*_2_ relaxometry to obtain such a measure. Here we evaluate the reproducibility and *B*_1_ sensitivity of IDEAL‐CPMG *T*_2_‐water and fat fraction (f.f.) values in healthy subjects, and demonstrate the potential of the method to quantify *T*_2_‐water variation in diseased muscle displaying varying degrees of fatty infiltration.

The calf muscles of 11 healthy individuals (40.5 ± 10.2 years) were scanned twice at 3 T with an inter‐scan interval of 4 weeks using IDEAL‐CPMG, and 12 patients with hypokalemic periodic paralysis (HypoPP) (42.3 ± 11.5 years) were also imaged. An exponential was fitted to the signal decay of the separated water and fat components to determine *T*_2_‐water and the fat signal amplitude muscle regions manually segmented.

Overall mean calf‐level muscle *T*_2_‐water in healthy subjects was 31.2 ± 2.0 ms, without significant inter‐muscle differences (*p* = 0.37). Inter‐subject and inter‐scan coefficients of variation were 5.7% and 3.2% respectively for *T*_2_‐water and 41.1% and 15.4% for f.f. Bland–Altman mean bias and ±95% coefficients of repeatability were for *T*_2_‐water (0.15, −2.65, 2.95) ms and f.f. (−0.02, −1.99, 2.03)%. There was no relationship between *T*_2_‐water (ρ = 0.16, *p* = 0.07) or f.f. (ρ = 0.03, *p* = 0.7761) and *B*_1_ error or any correlation between *T*_2_‐water and f.f. in the healthy subjects (ρ = 0.07, *p* = 0.40). In HypoPP there was a measurable relationship between *T*_2_‐water and f.f. (ρ = 0.59, *p* < 0.001).

IDEAL‐CPMG provides a feasible way to quantify *T*_2_‐water in muscle that is reproducible and sensitive to meaningful physiological changes without *post hoc* modeling of the fat contribution. In patients, IDEAL‐CPMG measured elevations in *T*_2_‐water and f.f. while showing a weak relationship between these parameters, thus showing promise as a practical means of quantifying muscle water in patient populations.

**Abbreviations Used**
ANOVA: analysis of variance
C.I.: confidence interval
CoV: coefficient of variation
CPMG: Carr–Purcell–Meiboom–Gill
DP: deep posterior compartment
f.f.: fat fraction
GRE: gradient echo
HypoPP: hypokalemic periodic paralysis
IDEAL: iterative decomposition with echo asymmetry and least‐squares estimation
LG: lateral gastrocnemius
MG: medial gastrocnemius
PL: peroneus longus
ROI: region of interest
STIR: short‐tau inversion recovery
TA: tibialis anterior
S: soleus
s.d.: standard deviation
TI: time to inversion

## 1 INTRODUCTION

The accurate quantification of muscle‐water transverse magnetization relaxation time (*T*_2_) as a potential treatment‐trial outcome measure is an important challenge.^1^ As trials of important new therapies in muscle diseases commence, there is a need for outcome measures to quantify disease progression and treatment effects that are more responsive than existing functional tests.^2^ Muscle fat infiltration and edema are both common pathological manifestations in neuromuscular diseases, and may in principle occur concurrently or independently; changes in muscle‐water *T*_2_ (*T*_2_‐water) may thus be hypothesized to occur prior to, independently of, or in conjunction with muscle infiltration by fat. *T*_2_ measurement methods that do not discriminate between fat and water signal contributions yield a combined *T*_2_ value that has been termed the global *T*_2_.^1^ The *T*_2_ of fat is longer than *T*_2_‐water in healthy muscle, and therefore, unless the fat and water signal contributions can be separated, increased fat content and increased *T*_2_‐water both elevate the apparent global *T*_2_, complicating the interpretation of such a finding. Methods to obtain muscle *T*_2_‐water independently of fat content are therefore needed in order to assess the potential of *T*_2_‐water as a marker of disease progression or treatment response providing pathological specificity beyond that that provided by muscle‐fat measurement alone.

Conventionally, *T*_2_ is measured in MRI by collecting multiple spin‐echo images and fitting the signal decay with echo time (TE) to a theoretical model of the expected signal behavior. The respective independent contributions of fat and water to the decay signal can be obtained by two approaches: by modifying the acquisition sequence to provide selective relaxation‐time or chemical‐shift based signal separation, where the fat signal is suppressed with spectrally selective saturation or inversion recovery methods leaving a signal that is dominated by the water component,^3^ or by attempting to fit an appropriate multi‐component model to the combined signal time course, assuming the measured signal is the sum of two or more independent decay functions attributable to water and fat.^4^, ^5^ This latter method may be augmented by chemical‐shift fat–water signal separation techniques such as three‐point Dixon imaging implemented in a separate additional acquisition, providing an independent measurement of the fat–water ratio that can be used to constrain a multi‐exponential fit to the non‐fat‐suppressed spin‐echo decay.^6^ The IDEAL‐CPMG pulse sequence^7^ combines IDEAL (iterative decomposition with echo asymmetry and least‐squares estimation) fat–water chemical shift separation^8^ with Carr–Purcell–Meiboom–Gill (CPMG) multi‐spin‐echo *T*_2_ measurement, thus permitting independent measurements of *T*_2_‐water and *T*_2_‐fat in a single acquisition.

To date, the feasibility of IDEAL‐CPMG muscle *T*_2_ relaxometry has been demonstrated in five healthy volunteers and in a single representative patient, with methodological validity evaluated *in vitro*.^7^ Here we evaluate the performance of IDEAL‐CPMG *T*_2_‐water relaxometry in healthy and diseased muscle. We present normative 3 T *T*_2_‐water values for calf‐level muscles, and assess in a group of healthy adults scan–scan stability, and the relationship between *T*_2_‐water estimates and transmit *B*_1_ variation. This relationship is important because *B*_1_ variation has been identified as a major determinant of *T*_2_‐water estimation accuracy in multi‐component modeling of conventional CPMG data.^4^ Finally, in a cohort of patients with hypokalemic periodic paralysis (HypoPP),^9^, ^10^ a muscle ion channelopathy causing periodic attacks of weakness in which patients present with a spectrum of both edematous and fatty‐muscle pathology, the relationship between measured *T*_2_‐water and muscle fat content is investigated to determine how effectively IDEAL‐CPMG decouples *T*_2_‐water measurements as fat content varies.

## 2 METHODS

### 2.1 Image acquisition

Both calves were imaged at 3 T (Siemens TIM Trio, Erlangen, Germany) in a feet‐first supine position with surface matrix coils for signal reception. The IDEAL‐CPMG pulse sequence was implemented (TR = 3000 ms, 3 × 5 mm slices, 10 mm gap, 192 × 96 matrix, 41 × 20.5 cm^2^ field of view (FOV)) with three gradient‐echo (GRE) shifts at (−1.02, 0.61, 2.25) ms around each of 16 spin echoes with spin‐echo times (TE) from 12 ms with a 12 ms interval with a bandwidth of 2003 Hz/pixel. The central slice was prescribed at a fixed distance of 15 cm below the knee joint in all cases. To independently assess muscle *T*_2_‐weighted signal change patterns, short‐tau inversion recovery fat‐suppressed (STIR) images (TR/TE/TI = 5500/56/220 ms, 9 × 10 mm slices, 256 × 120 matrix) were also acquired. A high‐resolution GRE image was acquired to provide a reference for muscle segmentation (TR/TE = 100/3.45 ms, flip angle =10°, matrix 512 × 240, FOV 44 × 20.6 cm^2^). *B*_1_ maps were acquired with the double angle method^11^ (TE/TR = 11/7000 ms, FOV 44 × 20.6 cm^2^, 40 × 10 mm slices, nominal flip angles 60° and 120°), with *B*_1_ error expressed as the percentage deviation of the actual flip angle from that nominally prescribed.

11 healthy volunteers (eight male, mean age ± standard deviation (s.d.) 40.5 ± 10.2 y, range 26.5–61.3 y) with no known muscle pathology were scanned at baseline (Scan A) and again after four weeks (Scan B) (mean interval 27.4 ± 3.5 days) to assess the stability of muscle *T*_2_ measurements. 12 HypoPP patients (nine male, 42.3 ± 11.5 y, range 23.2–58.5 y) were scanned once with the same acquisition protocol as the healthy subjects. Inclusion criteria were genetically confirmed diagnosis with mutations in the CACNA1S gene or SCN4A gene and clinical evidence of active disease, with either patient report of attacks of weakness, or progressive fixed weakness on examination. Patients had a mean disease duration of 30.8 ± 11.4 y, range 15–53 y. At the time of scanning, nine patients were on regular medication, one patient on as‐required medication and two patients on no medication for HypoPP. Most commonly used medications were Sando‐K in eight patients and acetazolamide in five patients.

### 2.2 Data processing and analysis

**Figure 1:**
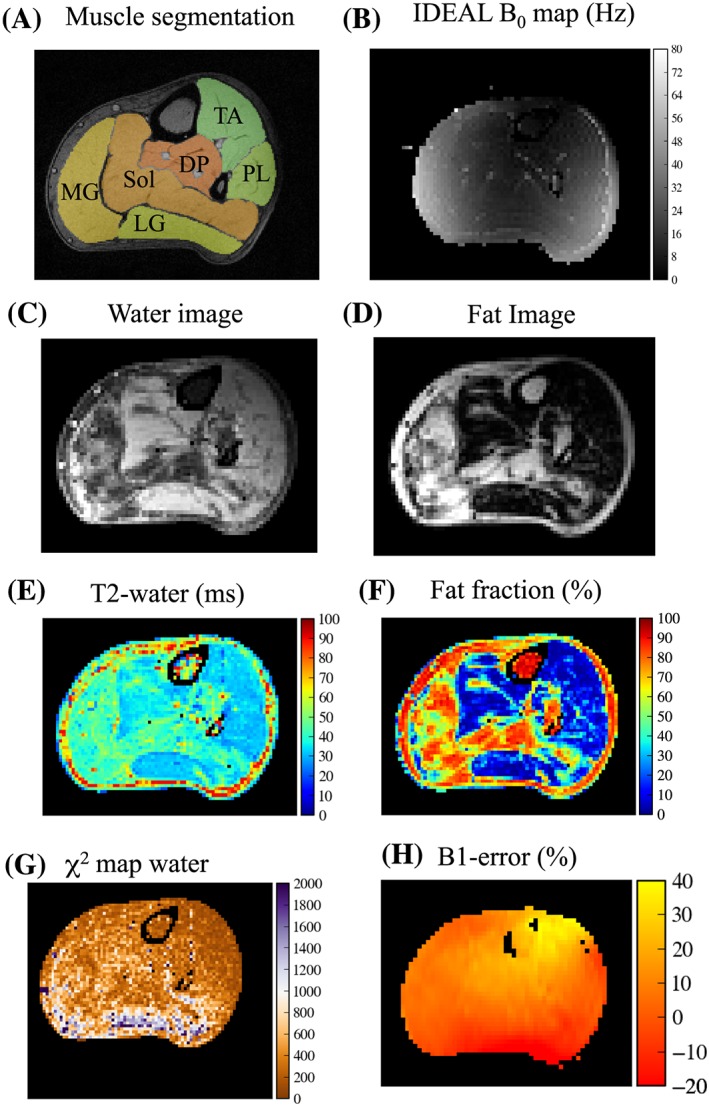
A, Example high‐resolution GRE image of a volunteer with muscle segmentations overlaid. B–F, The separation of water and fat with IDEAL‐CPMG in a 53‐year old patient with HypoPP. B, *B*_0_ offset map obtained in the IDEAL decomposition. C,D, The raw signal is separated into the water (C) and fat (D) components with the IDEAL algorithm to produce 16 such pairs of images for each spin echo. Mono‐exponential functions are fitted to the signal decays. E, The *T*_2_ of the water component (*T*_2_‐water) is determined from the decay rate of the signal fitted to the water component. F, The f.f. is calculated as the ratio of the fitted proton‐density amplitudes at *t* = 0. G, The *χ* 2 goodness of fit for the water component. H, The *B*_1_‐error map

Imaging data were exported from the scanner and processed offline using the Python programming language (www.python.org) and Wolfram Mathematica 10 (Champaign, IL, USA). A radiologist performed manual muscle segmentation on the GRE images using the ITK‐SNAP software,^12^ outlining six separate muscles (tibialis anterior (TA), peroneus longus (PL), lateral gastrocnemius (LG), medial gastrocnemius (MG) and soleus (S), and a deep posterior compartment (DP) (encompassing tibialis posterior, flexor digitorum longus and flexor hallucis longus) in each limb at a single level on the middle slice (Figure 1A)), excluding major vessels and nerves. The segmentations were resampled to match the IDEAL‐CPMG acquisition matrix and the borders eroded to reduce contamination from subcutaneous fat at the region of interest (ROI) edges. The IDEAL fat–water separation algorithm was implemented^13^ with the NumPy Python package, using a seven‐peak model of the fat spectrum^7^ to derive fat and water‐only images for each spin echo, and a map of static field (*B*_0_) variation.

To estimate tissue *T*_2_ values and proton densities, mono‐exponential decay functions were fitted to the fat and water signals separately using a least‐squares Levenberg–Marquardt algorithm. In all cases the first echo was excluded from the fit to ensure consistent stimulated‐echo coherence contributions. The amplitude (*A*), *T*_2_ and signal offset (*c*) were determined for the water (w) and fat (f) components respectively on a pixel‐wise basis by fitting to the function *S* = *A* exp (‐*t*/*T*2) + *c*. The unreduced *χ* 2 sum of squares was used to assess goodness of fit.

To determine the fat to MRI‐visible proton‐density ratio (apparent f.f.) the sum of the total signal extrapolated to TE = 0 was used in order to minimize *T*_2_‐weighting bias^7^ such that f.f. = *A*_f_/(*A*_w_ + *A*_f_).

The Scan A–Scan B reproducibility for the healthy subjects was evaluated using the two s.d. limits of agreement and intra‐class correlation coefficients, with signed rank tests for paired comparisons. The individual source data points for this analysis were obtained as the ROI mean of each muscle segmentation applied to the respective IDEAL‐CPMG *T*_2_ maps. Coefficients of variation (CoVs) were calculated according to Reference 14. Inter‐muscle variation was investigated by analysis of variance (ANOVA) with a significance level of 0.05. Left–right differences in *B*_1_ were compared on a muscle‐wise basis with paired *t*‐tests. The relationship between *T*_2_‐water and f.f. was evaluated with Spearman rank coefficients, with *p* < 0.05 considered significant. Patient–control group differences were evaluated using the Mann–Whitney test.

## 3 RESULTS

### 3.1 IDEAL‐CPMG fat–water separation and fitting

IDEAL‐CPMG successfully separated the water and fat signal for each spin echo. Examples of IDEAL‐CPMG fat–water separation and fitting are shown in Figure 1. The *B*_0_ map derived from the IDEAL algorithm is shown in Figure 1B, with the accompanying water and fat separated images in Figure 1C,D respectively. Figure 1E,F shows the derived *T*_2_‐water and f.f. maps for this subject. Figure 1G,H shows the *χ* 2 map for the water component and the *B*_1_‐error map respectively.

**Figure 2:**
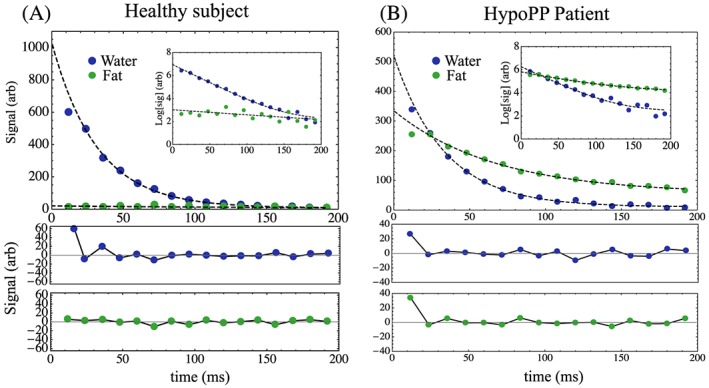
Mono‐exponential fits and accompanying residual plots for the water and fat signals for a single pixel in the S muscle of a healthy subject and a patient. Fits are expressed as dashed lines. Insets denote the natural logarithm of the data and fit. The residual differences between the signal and the fits are plotted in the panels beneath. The first sampled echo is not included in the fit optimization function. Fitted parameters were A, water (*A*_w_ = 1020.5, *T*_2w_ = 31.6 ms, *c*_w_ = 8.1), fat (*A*_f_ = 18.6, *T*_2f_ = 200 ms, *c* = 1.9), f.f. = 1.8%, and B, water (*A*_w_ = 509.6, *T*_2w_ = 33.1 ms, *c* = 10.8), fat (*Af* = 279.4, *T*_2f_ = 67.0 ms, *c* = 55.3) and f.f. = 35.4%

Examples of mono‐exponential fits to the *T*_2_‐decay signals with accompanying residual differences between the fits and the data are shown in Figure 2 for representative single pixels in the soleus muscles of a healthy subject with low muscle‐fat content, yielding f.f. of 1.8% (Figure 2A), and a patient with f.f. of 35.4% (Figure 2B).

### 3.2 *T*_2_‐water and f.f. in healthy subjects

The healthy volunteer data are tabulated by muscle in Table 1. The mean (±s.d.) *T*_2_‐water across all muscles in Scan A was 31.2 ± 2.0 ms. ANOVA did not reveal any significant inter‐muscle differences (*p* = 0.37). The greatest variance was observed in the lateral and medial gastrocnemii muscles, attributable to two subjects displaying transient elevated water content in these muscles.

**Table 1:** The group mean and s.d. values for *T*_2_‐water and f.f. for each muscle examined in the healthy subjects. Scan A and Scan B denote the 4 week separated scans. Mean values across all muscles are given in the final row. CoVs for inter‐subject and inter‐scan differences are given (expressed as a percentage of the respective mean value)

**Muscle**	**T2‐water (ms)**				**f.f. (%)**			
	**Mean**		**CoV (%)**		**Mean**		**CoV (%)**	
	**Scan A**	**Scan B**	**Inter‐subject**	**Inter‐scan**	**Scan A**	**Scan B**	**Inter‐subject**	**Inter‐scan**
**Right TA**	30.4 ± 1.3	30.3 ± 1.0	3.7	1.3	3.8 ± 0.9	3.6 ± 1.0	24.5	10
**Right PL**	30.3 ± 0.9	30.5 ± 1.1	3.2	2.3	6.3 ± 1.5	6.2 ± 1.9	26.3	12.3
**Right LG**	31.8 ± 2.9	31.1 ± 2.0	7.9	4.8	5.1 ± 1.8	5.8 ± 2.4	41.0	26.2
**Right MG**	31.2 ± 2.2	30.4 ± 1.2	5.7	4.2	4.3 ± 1.2	4.2 ± 1.3	27.9	6.3
**Right S**	32.0 ± 1.1	32.0 ± 0.8	3.0	2.3	4.5 ± 1.5	4.3 ± 1.4	31.3	6.1
**Right DP**	31.3 ± 1.0	31.5 ± 1.2	3.4	2.0	4.0 ± 2.1	3.9 ± 1.7	45.7	16.7
**Left TA**	30.8 ± 0.7	31.1 ± 1.0	2.7	2.0	3.5 ± 1.0	3.5 ± 0.7	23.8	12.9
**Left PL**	30.3 ± 1.6	30.7 ± 1.9	5.7	2.6	5.8 ± 2.2	5.4 ± 2.0	35.1	10.5
**Left LG**	31.1 ± 3.3	30.4 ± 2.0	8.7	4.5	4.2 ± 1.4	5.1 ± 1.7	37.8	31.2
**Left MG**	31.6 ± 3.4	30.9 ± 2.3	9.0	4.1	5.4 ± 3.1	5.1 ± 2.8	53.3	8.9
**Left S**	31.5 ± 1.2	31.4 ± 1.0	3.3	2.7	4.5 ± 2.0	4.5 ± 2.0	44.1	7.1
**Left DP**	31.9 ± 0.9	32.0 ± 1.6	4.1	2.8	3.7 ± 1.3	3.3 ± 1.1	32.3	14.4
**Mean all muscles**	**31.2 ± 2.0**	**31.0 ± 1.6**	**5.7**	**3.2**	**4.6 ± 1.9**	**4.6 ± 1.9**	**41.1**	**15.4**

Mean f.f. across all muscles in Scan A was 4.6 ± 1.9%. The individual muscle with the highest mean f.f. was the PL, and that with the lowest the TA (Table 1), with ANOVA indicating that inter‐muscle f.f. differences were significant (*p* = 0.004).

### 3.3 Stability of *T*_2_‐water and f.f. measured in healthy subjects

**Figure 3:**
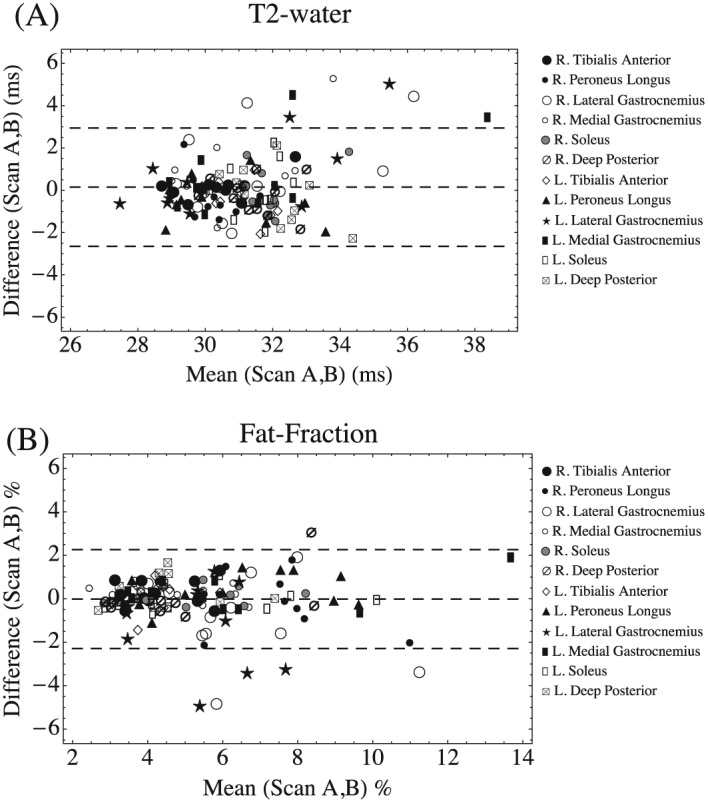
Bland–Altman plots for 4 week repeat measurements (Scans A and B) of *T*_2_‐water and f.f. in the healthy subjects. Dashed lines represent the mean bias and ±2 s.d. limits of agreement (0.15, −2.65, 2.95) ms and (0.02, −1.99, 2.03)% respectively. Symbols represent the individual muscles. No systematic clustering by muscle can be observed

Table 1 gives the CoVs for inter‐subject and 4 week inter‐scan differences for each muscle and for all muscles combined. *T*_2_‐water inter‐subject variation was higher than the inter‐scan variation (5.7% compared with 3.2%). Similarly, inter‐subject f.f. variation (41.1%) was higher than the inter‐scan variation (15.4%). Overall intra‐class correlation coefficients for the 4 week scan differences were 0.70 (*T*_2_‐water) and 0.86 (f.f.), with paired sign‐rank tests for 4 week differences non‐significant for all muscles (*p* = 0.92 *T*_2_‐water and *p* = 0.12 f.f.) Bland–Altman plots for all muscles are shown for (A) *T*_2_‐water and (B) f.f. in Figure 3 with respective mean bias and limits of agreement of (0.15, −2.65, 2.95) ms and (−0.02, −1.99, 2.26)% depicted by dashed lines.

**Figure 4:**
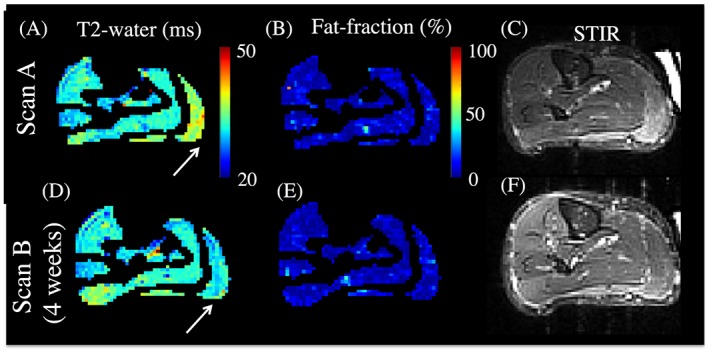
Elevated *T*_2_‐water in the gastrocnemius muscles of a healthy subject. A, The *T*_2_‐water map displays elevated values in the LG and MG muscles (white arrow). B,C, The f.f. map does not display any abnormality (B) but the STIR images confirm the genuine fluid changes (C). D–F, After 4 weeks the region of elevated *T*_2_‐water (D) has decreased, with corresponding f.f. map (E) and STIR image (F)

Figure 4 shows a healthy individual at Scan A and Scan B, demonstrating an initially elevated right MG *T*_2_‐water (Figure 4A) that had returned to a normal level after 4 weeks (Figure 4D). There was no appreciable change in f.f. (Figure 4B,E), while MG and LG muscle STIR image hyper‐intensity at Scan A (Figure 4C) corroborates the presence of true water‐related changes in this muscle at that time.

### 3.4 *T*_2_‐water versus f.f. in healthy subjects

**Figure 5:**
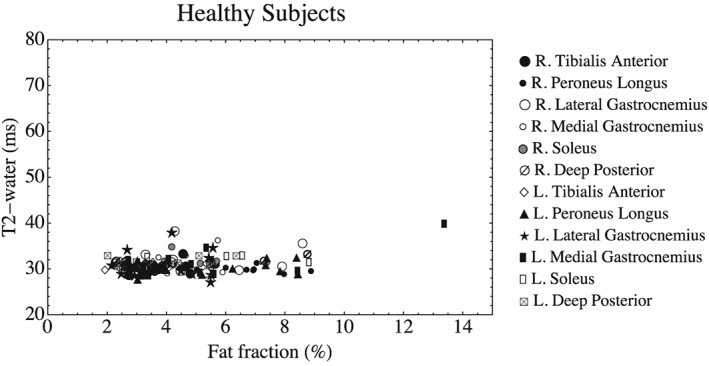
*T*_2_‐water plotted against IDEAL f.f. for the 11 healthy subjects (Scan A). Symbols denote the muscle ROI. There is no linear gradient denoting a relationship between f.f. and *T*_2_‐water in these subjects

Mean muscle *T*_2_‐water is plotted against f.f. for each muscle in the healthy subjects in Figure 5. There was no significant correlation between *T*_2_‐water and f.f. (Spearman's ρ =0.07, *p* = 0.40).

### 3.5 Influence of *B*_1_ transmit homogeneity

**Figure 6:**
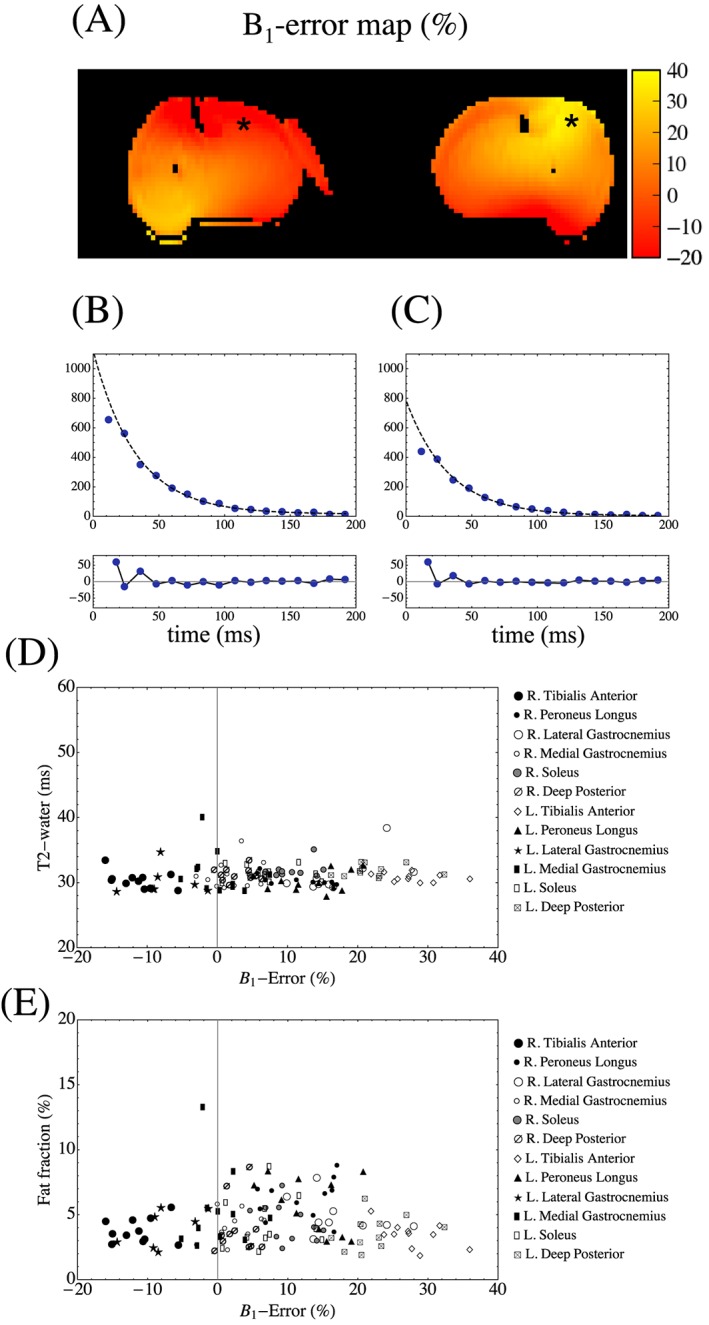
A, Example *B*_1_‐error map in a healthy subject, scaled to the range of −20 to +40% deviation from the prescribed flip angle. B,C, *T*_2_‐water signal for the pixels in different *B*_1_ regions denoted by the asterisks in A, yielding fits of *A*_w_ = 1104.5, *T*_2w_ = 32.6 ms, *c*_w_ = 16.1 and *A*_w_ = 782.0, *T*_2w_ = 32.4 ms, *c*_w_ = 4.2 respectively. D,E, The mean *T*_2_‐water and f.f. against *B*_1_ error for each muscle in the healthy subjects. Spearman's rank coefficients were non‐significant, indicating that there was no measurable relationship between these measured quantitative parameters and *B*_1_

An example of a *B*_1_‐error map in a healthy subject is shown in Figure 6A, with the *B*_1_‐error color‐scale range of −20 to +40% chosen to emphasize *B*_1_ deviation across the image. *T*_2_‐water signal decays are shown for two different pixel regions in Figure 6B,C, denoted by asterisks in Figure 6A. The relationship between ROI‐mean *T*_2_‐water and *B*_1_ error is plotted for all healthy subject ROIs in Figure 6D and between f.f. and *B*_1_ error in Figure 6E on a muscle‐by‐muscle basis. There was no relationship between *T*_2_‐water and *B*_1_ error (Spearman's ρ 0.16, *p* = 0.07) and no significant correlation between f.f. and *B*_1_ error (Spearman's ρ 0.03, *p* = 0.77).

### 3.6 *T*_2_‐water versus f.f. in HypoPP patients

The overall mean *T*_2_‐water and f.f. in the HypoPP patient group were 34.5 ± 6.6 ms and 14.7 ± 16.9% respectively, and both were elevated compared with the healthy volunteer group (*p* < 0.001). ROI mean *T*_2_‐water and f.f. are given for the left‐limb individual muscles of the 12 patients in Table 2. ANOVA did not indicate significant inter‐muscle differences for *T*_2_‐water (*p* = 0.40) or f.f. (*p* = 0.38).

**Table 2:** Mean *T*_2_‐water and f.f. values for the left limb muscles in the 12 HypoPP patients studied

**Patient No**	**Left TA**		**Left PL**		**Left LG**		**Left MG**		**Left S**		**Left TP**	
	**T2‐water (ms)**	**f.f. (%)**	**T2‐water (ms)**	**f.f. (%)**	**T2‐water (ms)**	**f.f. (%)**	**T2‐water (ms)**	**f.f. (%)**	**T2‐water (ms)**	**f.f. (%)**	**T2‐water (ms)**	**f.f. (%)**
**1**	29.8 ± 2.7	4.1 ± 5.6	29.3 ± 1.7	8.3 ± 7.4	31.9 ± 2.1	4.1 ± 6.5	31.5 ± 1.4	3.4 ± 4.6	33.3 ± 1.9	4.6 ± 4.3	30.9 ± 1.6	4.8 ± 4.1
**2**	31.2 ± 1.9	3.7 ± 3.0	31.9 ± 5.1	11.8 ± 16.1	35.7 ± 1.7	5.0 ± 11.3	33.6 ± 2.0	3.9 ± 3.3	33.6 ± 3.9	3.1 ± 3.0	31.9 ± 2.4	9.2 ± 8.2
**3**	32.3 ± 3.5	10.3 ± 12.3	32.0 ± 3.6	17.7 ± 12.9	33.6 ± 2.3	15.2 ± 11.4	43.9 ± 9.1	85.2 ± 6.4	38.1 ± 6.4	33.3 ± 28.8	33.8 ± 5.8	27.4 ± 25.1
**4**	33.3 ± 2.8	12.0 ± 11.1	31.3 ± 1.8	13.2 ± 8.4	51.7 ± 9.0	36.9 ± 17.3	41.8 ± 4.8	33.6 ± 14.0	40.6 ± 2.8	19.1 ± 12.2	38.5 ± 6.9	13.7 ± 11.1
**5**	32.0 ± 1.9	4.9 ± 4.0	31.6 ± 3.7	16.2 ± 15.6	51.5 ± 8.7	38.8 ± 21.2	35.4 ± 3.8	34.2 ± 17.9	34.0 ± 2.6	9.6 ± 8.1	32.3 ± 3.0	8.2 ± 8.6
**6**	47.0 ± 10.1	50.1 ± 21.9	45.4 ± 6.9	67.0 ± 13.8	68.3 ± 7.3	51.9 ± 4.5	47.7 ± 7.3	67.5 ± 11.1	38.6 ± 5.1	25.5 ± 16.9	37.4 ± 3.8	25.3 ± 12.3
**7**	31.6 ± 1.8	7.3 ± 6.7	31.9 ± 1.6	38.6 ± 9.0	29.7 ± 1.4	8.5 ± 5.5	34.5 ± 2.8	15.0 ± 7.4	33.8 ± 2.1	19.6 ± 10.9	31.8 ± 2.6	8.5 ± 6.2
**8**	31.5 ± 3.2	8.1 ± 9.0	31.6 ± 2.4	12.4 ± 9.6	33.6 ± 3.7	9.7 ± 8.1	31.9 ± 3.1	11.1 ± 15.0	33.8 ± 4.3	17.8 ± 20.6	38.0 ± 6.1	77.3 ± 22.4
**9**	29.2 ± 1.3	5.0 ± 6.2	32.1 ± 2.3	12.1 ± 11.0	32.3 ± 1.8	3.6 ± 2.7	31.3 ± 1.8	8.3 ± 7.9	31.7 ± 1.5	8.1 ± 6.6	30.5 ± 3.6	6.3 ± 6.3
**10**	33.0 ± 2.6	3.8 ± 4.2	31.3 ± 1.6	4.6 ± 5.1	35.4 ± 4.9	3.3 ± 2.3	32.6 ± 2.5	5.8 ± 6.0	36.1 ± 2.2	4.1 ± 4.6	31.8 ± 1.7	4.6 ± 6.7
**11**	30.0 ± 1.4	3.7 ± 5.5	31.8 ± 3.0	6.6 ± 5.7	29.5 ± 1.4	5.0 ± 2.5	29.2 ± 1.8	3.5 ± 2.8	31.2 ± 1.8	2.8 ± 2.0	30.4 ± 2.6	2.5 ± 1.9
**12**	29.6 ± 2.6	4.3 ± 3.0	28.0 ± 3.7	9.8 ± 8.8	33.2 ± 4.1	7.9 ± 5.0	33.2 ± 1.3	2.9 ± 2.4	33.5 ± 3.0	3.6 ± 2.7	32.8 ± 4.4	3.8 ± 3.1

### 3.7 Relationship between *T*_2_‐water and f.f. in patients

**Figure 7:**
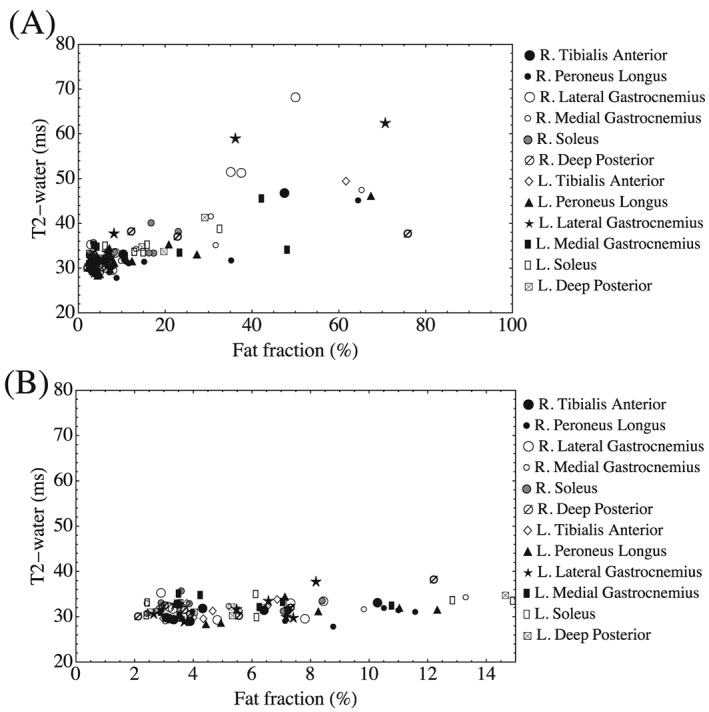
A, Mean *T*_2_‐water plotted against mean f.f. for all muscle ROIs in the HypoPP patients. B, The same data plotted in the range 0–15% f.f. There are examples of highly elevated f.f. without corresponding *T*_2_‐water elevation and cases where both are elevated simultaneously

A plot of individual muscle ROI mean *T*_2_‐water versus f.f. is shown for the patient group in Figure 7, where there was a greater spread of both *T*_2_‐water and f.f. (Table 2) than in healthy volunteers. The equivalent correlations, gradients and intercepts were (ρ = 0.59, *p* < 0.001), 0.31 ms/% (95% confidence interval (C.I.) 0.26–0.36 ms/%) and 30.3 ms (95% C.I. 29.3–31.3 ms) respectively. Figure 7B shows the same data as in A in the range 0–15% f.f.

**Figure 8:**
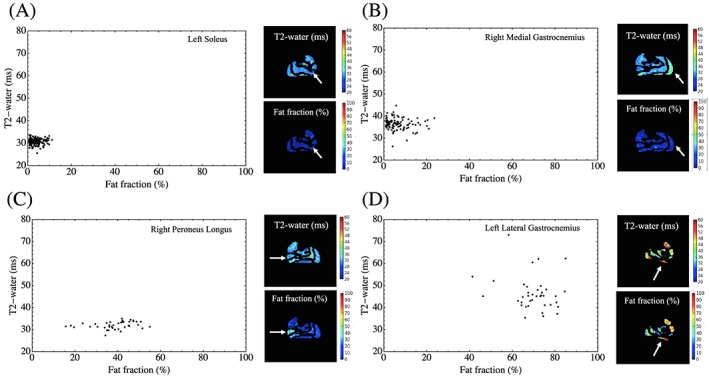
Pixelwise plots of *T*_2_‐water versus f.f. in individual subjects. A, Plot of pixel values in the left soleus muscle of a healthy subject that displays neither *T*_2_‐water nor f.f. elevation. B, Pixel values in the right MG muscle in a healthy subject illustrating elevation of *T*_2_‐water without substantial fat infiltration. C, f.f. in the PL muscle in a HypoPP patient is substantially elevated while *T*_2_‐water values remain within the normal range. D, Both *T*_2_‐water and f.f. are elevated in the left LG muscle of a HypoPP patient. White arrows indicate the respective muscle plotted in each case

Figure 8 shows individual pixel plots to illustrate how the range of scatter in Figure 7 arises from the behavior in given individual muscles, demonstrating the pixel‐level relationship between *T*_2_‐water and f.f. in a number of different conditions. Figure 8A,B shows healthy volunteer S and MG muscles respectively where no (A) and moderate (B) *T*_2_‐water elevations are observed without corresponding increase in measured f.f. In Figure 8C there are substantial increases in f.f. of the PL muscle of a HypoPP patient while the *T*_2_‐water values remain in the normal range. In the LG muscle of the HypoPP patient in Figure 8D, both quantities are elevated in a heterogeneous manner.

## 4 DISCUSSION

In this work we aimed to address practical and technical considerations concerning the use of IDEAL‐CPMG to quantify the *T*_2_ of muscle water in the presence of fat infiltration, and demonstrate the sensitivity of the measurements in a group of healthy subjects and in a representative disease. We observe that IDEAL‐CPMG imaging provides stable measures of calf‐level muscle *T*_2_‐water in repeated measurements in healthy individuals (Figure 3 and Table 1), with sufficient sensitivity to detect minor *T*_2_ elevations, even in healthy subjects (Figure 4) and without correlation with transmit field *B*_1_ inhomogeneity (Figure 6). The method can determine *T*_2_‐water in a variety of muscle tissue states. In terms of muscle ROI mean values, at low f.f. levels *T*_2_‐water is not influenced by fat infiltration in either healthy subjects or patients (Figure 5, Figure 7). When fat is present in greater quantities in patients, mean muscle *T*_2_‐water is elevated in some cases (Figure 7). At the individual pixel level, a range of scenarios of *T*_2_‐water and f.f. relationships can be discriminated, as illustrated by the pixel‐wise plots in individual muscles (Figure 8), which may contribute to the observed overall ROI mean effects.

There is an important need to be able to quantify the water component of muscle in neuromuscular conditions, which this work aims to address. Effective muscle water *T*_2_ measurement is a valuable tool for probing normal muscle functional physiology and for tracking disease processes, which may be amenable to therapeutic intervention. It is well known that exercise challenges elevate the MRI‐visible water load in normal muscle.^15^ In conditions where the normal function of muscle is affected by molecular dystrophic deficiencies^16^ or deficiencies in ion channel transport,^17^ water changes such as edema and inflammation are important markers of disease activity. Crucially, these can occur before significant fat infiltration or atrophy is present, and alterations in water load may offer markers indicating successful application of a therapy to its target. Indeed, for novel therapies that may involve exercise itself,^18^ quantifying such muscle changes under these circumstances is essential. Therefore methods to detect water changes sensitively at an early stage by quantifying *T*_2_, in early disease and then progressively as fat begins to encroach simultaneously with evolving water changes, have been receiving increased attention. This work evaluates IDEAL‐CPMG as a practical method for measuring muscle water *T*_2_ in the presence of fat.

### 4.1 *T*_2_‐water and f.f. in healthy subjects

The mean *T*_2_‐water obtained in healthy lower‐limb muscles, 31.2 ± 2.0 ms, was consistent with values recently reported in other 3 T studies using different strategies to obtain fat‐independent *T*_2_‐water in thigh muscles,^19^, ^20^ and with a 32 ms‐centered *T*_2_ component observed in the soleus muscle from a localized 1000 TE CPMG acquisition.^21^ Using standard non‐fat‐suppressed CPMG MRI, Forbes et al.^22^ reported a soleus *T*_2_ of 32.2 ± 1.9 ms in healthy boys, compared with a slightly lower value (28.1 ± 0.81 ms) using a spectrally resolved multi‐echo MRS method.

The distribution of *T*_2_‐water values in healthy subjects was low compared with plausible increases due to disease (Table 2), indicating that small pathological perturbations should be readily detectable with IDEAL‐CPMG. In circumstances of disease or exercise‐driven *T*_2_‐water changes, defining the normal range as 1.96 s.d. around the mean would imply an upper threshold of 35.1 ms for overall *T*_2_‐water abnormality. The pattern of highest f.f. in PL and lowest f.f. in TA is consistent with previously reported patterns.^23^, ^24^

### 4.2 Stability and sensitivity in healthy subjects

An important property of any quantitative method is good reproducibility under typical clinical acquisition conditions, which we assessed here by repeat scanning of healthy subjects at a 4 week interval. The overall indices of reproducibility in healthy subjects were high (indicated by the Bland–Altman limits of agreement (Figure 3) and CoVs (Table 1). Moreover, sensitivity was sufficient to observe a genuine serendipitous change in *T*_2_‐water in isolated muscles in a healthy subject over a 4 week period (Figure 4). The origin of these changes is not clear, although transient sequelae of a physical exertion, resolved over the timescale of weeks, seem a likely cause. Importantly, reference to qualitative normalized STIR images corroborated these changes, providing independent qualitative verification of *T*_2_‐water elevations representing and quantifying genuine fluid‐driven effects in this healthy individual. The sensitivity of IDEAL‐CPMG to detect small *T*_2_‐water changes such as those associated with everyday activity is an important advantage, and highlights the need to control for such factors in patient studies where both pathology‐ and everyday‐activity‐related effects may induce parallel *T*_2_‐water changes.

When assessing the stability of *T*_2_‐water measurements, it is instructive to consider the changes expected due to disease, exercise or injury. In the case of changes expected in disease, mean skeletal muscle *T*_2_‐water increases between patient and control groups have been reported to be around 10–15% in Duchenne muscular dystrophy^22^, ^25^, ^26^ and Pompe disease.^27^ The metabolic sequelae of exercise immediately before measurement may increase muscle *T*_2_ by around 5 ms (16%),^28^ with recovery within 45 minutes to baseline values.^29^ Eccentric exercise of sufficient intensity to result in muscle injury causes longer‐term *T*_2_ changes, with for example a 2 ms increase detectable after 24 h,^30^ rising to a peak value up to 20 ms above baseline 3–5 days after injury.^31^

### 4.3 *B*_1_ insensitivity

A common finding in quantitative MRI is errors arising under non‐ideal measurement conditions such as static or RF magnetic field homogeneity. Such errors must be understood or corrected to permit proper interpretation of observed variations. In this work we used the double angle method to measure the *B*_1_‐transmit RF inhomogeneity. The *B*_1_ variation (Figure 6A) over the imaging slice was marked, and typical of that commonly observed in calf muscles using conventional circularly polarized transmission at 3 T.^32^ However, our *T*_2_‐water and f.f. measurements did not demonstrate any significant dependence on *B*_1_ error (Figure 6D,E), indicating for the first time that the IDEAL‐CPMG method is fairly robust to the influence of transmit field variation. This is in contrast to findings reported when fitting multi‐exponential functions to non‐fat‐suppressed CPMG data that may be to some extent influenced by *B*_1_,^4^ and may represent a major advantage of IDEAL‐CPMG in this context. This may in part be a consequence of the performance of IDEAL fat–water separation being to a large extent independent of *B*_1_‐driven source‐image amplitude variations, whereas methods dependent on estimating both fat and water amplitudes, as well as their respective *T*_2_ values, by fitting a single multi‐component model to a combined CPMG decay signal, may result in both *B*_1_‐dependent apparent f.f. and *T*_2_ errors. The IDEAL‐CPMG approach may compliment other recently reported methods with reduced sensitivity to *B*_1_ effects by implicitly including *B*_1_ field in a multi‐parameter extended phase graph modeling of the signal decay.^20^

### 4.4 Measurements in HypoPP

The HypoPP patient group showed statistically elevated *T*_2_‐water compared with healthy subjects, as well as f.f. elevation (Table 1, Table 2), supporting the value of IDEAL‐CPMG muscle *T*_2_ as an index of pathology in this condition. 23.6% of the patient ROI mean *T*_2_‐water values were outside the 1.96 s.d. range of the healthy volunteers. The majority of the group mean muscle values in the HypoPP group, although elevated, were therefore not outside the range of normality. Although not observed in our data set, sensitivity to reductions in *T*_2_‐water may also be important in other neuromuscular conditions, particularly in the light of recent reports that measured *T*_2_ may in fact decrease in Duchenne muscular dystrophy.^23^, ^24^

HypoPP is an autonomic dominant disorder associated with mutations in the CACNA1S or SCN4A genes causing periodic serum potassium changes and muscle weakness without myotonia.^9^ The effect of treatment with acetazolamide over 4 weeks has been previously measured using STIR imaging.^33^ Fat‐suppressed *T*_2_‐weighted imaging has previously been used to investigate imaging characteristics in a group of Asian subjects with HypoPP and healthy controls before and after exercise.^34^ These reports of *T*2‐weighted contrast changes suggest that the ability to quantify *T*_2_ changes in HypoPP may provide an important tool for monitoring this condition.

### 4.5 Measuring *T*_2_‐water in the presence of fat

In skeletal muscle, myocellular fat replacement and changes in the intra‐ and extra‐myocellular water distribution can hypothetically occur independently or simultaneously within a tissue volume depending on the underlying disease process. There is no *a priori* assurance that a region of muscle displaying substantive fat infiltration will not also exhibit simultaneous related or independent water‐driven *T*_2_ changes. Indeed it seems probable that a given tissue volume undergoing active pathological processes is likely to encompass a spectrum of simultaneously evolving *T*_2_‐influencing features, both fat and water based. This should be considered when assessing the effectiveness of any technique that aims to measure *T*_2_‐water prolongation independently of fat, because the scenario of absolute independence may often be hypothetical; apparently associated *T*_2_‐water and f.f. increases may reflect genuine *T*_2_‐water elevation temporally correlated with f.f. as two facets of disease progression with physically distinct substrates. This is a situation in direct contrast to the apparent coupling of tissue *T*_2_ and f.f. obtained in a non‐separated global *T*_2_ measurement.

The relationship between mean muscle *T*_2_‐water and f.f. is shown for each of 12 muscles in the 12 patients in Figure 7, and for illustrative pixel‐wise examples in individual muscles in Figure 8. When f.f. is elevated (>5%), there are examples where *T*_2_‐water remains close to normal values (e.g. Figure 8C) and others where the *T*_2_‐water is substantially elevated (Figure 8B,D). Despite the range of physical scenarios presented in Figure 8, it is nonetheless conceivable that incomplete decoupling of water and fat could still be present to some extent due to residual *T*_1_ or *T*_2_ weighting or an inadequate model for the fat spectrum. In addition, since the apparent f.f. is strictly the fat–water ratio determined by the fitted amplitudes *A*_w_ and *A*_f_, any coupling between *A*_w_ and *T*_2_‐water will also contribute to bias in the f.f.–*T*_2_‐water relationship. It is not typical to find a neuromuscular disease model where either fat or water changes occur definitively in isolation of each other that would allow these scenarios to be fully tested, although further investigation of exercise‐based water changes in healthy subjects may provide further insight.

### 4.6 Alternative methods to determine *T*_2_‐water

Imaging methods that measure *T*_2_‐water while accounting for concurrent fat infiltration are predominantly based either on *post hoc* numerical modeling or on attempts to eliminate the fat signal at the point of acquisition. For numerical stability, *post hoc* processing usually requires an independent measure of the f.f., or *a priori* fat *T*_2_ values, to constrain the amplitudes of multi‐exponential fitting of the raw decay signal or by investigating multi‐exponential models with some fit parameters fixed based on an understanding of the system. Recently Marty et al. have successfully used a full extended phase graph approach to obtain *T*_2_‐water fitting to a more comprehensive model.^20^ Fat suppression techniques at the point of acquisition include several varieties of spectral fat pre‐saturation of multi‐echo *T*_2_‐weighted sequences. In contrast, in IDEAL‐CPMG the acquisition pulse sequence is designed to acquire separate *T*_2_‐decay signals at several GRE phase‐shifts so that chemical‐shift fat–water separation can be used to decouple the fat and water signals in post‐processing, allowing the fat and water signal decays to be captured independently in a single acquisition. Indeed, the fat signal can be discarded if quantifying water *T*_2_ is the primary interest.

Previous treatments of composite *T*_2_‐decay signals have in most cases considered these to comprise a linear sum of mono‐exponential decay functions representing water and various triglyceride lipid spectral components. When the water signal is available in isolation, as it is here, it is important to be confident that a mono‐exponential *T*_2_‐decay description is appropriate. The example mono‐exponential decay functions fitted to the representative single pixel data in Figure 2 described the signal well. However, no attempt was made in this work to establish a meaningful description of the time‐domain behavior of the fat signal. Rather, a pragmatic approach was used to estimate the proton‐density at *t* = 0 by fitting a mono‐exponential function to the fat signal decay. The spectrum of fat has multiple components,^35^ each with a different characteristic decay time.^4^ The dominance of a long *T*_2_‐decay component, which is often observed in the fat signal decay, is most likely ascribable to the large constant offset *c*_f_. For the current purpose, an exponential function allows determination of the *y*‐axis crossing *A*_f_ in order to estimate the fat–water proportion with minimal bias due to *T*_2_ weighting.

Volume‐localized MRS may also be a useful tool to obtain *T*_2_‐water unambiguously separated from lipid resonance signals, and future work correlating MRS and IDEAL‐CPMG findings may shed light on the origin of elevated muscle water *T*_2_ in the presence of fat.

### 4.7 Considerations for IDEAL‐CPMG implementation

A key advantage of IDEAL‐CPMG is that the f.f. and *T*_2_‐water are obtained in the same acquisition. Although obtaining fat–water ratios from a separate acquisition has been demonstrated to provide an effective way to constrain multi‐component fits,^6^ in contrast IDEAL‐CPMG does not require an alignment or registration step to ensure that fat and water pixels coincide, a process vulnerable to error due to subject motion or misalignment.

In common with most full CPMG‐based acquisitions, the slice coverage achieved in a given repetition time is limited by the long echo train and the consequent overall burden of RF energy absorption. The in‐slice spatial resolution is also limited for the same reasons, and the requirement to fit multiple GREs in the IDEAL‐CPMG implementation imposes further constraints. Nonetheless, this approach allows an overall survey of the muscle bulk at multiple levels with sufficient spatial resolution to quantitatively assess patterns of fat and fluid infiltration in all key lower‐limb muscles (Figure 1). 2D or 3D three‐point Dixon acquisitions may be better suited for specific measurements of f.f. where high spatial resolution is required, but IDEAL‐CPMG is unique in permitting both *T*_2_‐water and co‐localized f.f. determination, albeit at the cost of lower coverage.

The relatively low spatial resolution and restricted anatomical coverage in terms of the number of slices attainable within a reasonable TR are a potential limitation of the current implementation of the method. While these restrictions may be mitigated to a certain extent in the future by implementing further acceleration methods, they are likely to limit applications requiring high spatial resolution, such as in the pediatric population. Nevertheless, numerous studies have successfully demonstrated the responsiveness of MRI biomarkers in neuromuscular diseases with analyses based on large ROIs, or whole muscle cross‐section means, from only a single slice.

The acquisition bandwidth is necessarily higher in IDEAL‐CPMG than in standard CPMG acquisitions, resulting in decreased signal to noise using this method. The SNR in the left soleus muscle in the first GRE measured about the first spin echo was in the range 50–100. Standard CPMG acquisitions are capable of yielding substantially higher SNR, and this should be taken into consideration when evaluating the suitability of this method. While there was no evidence in the data we present of any clear relationship between regional SNR and precision of the *T*_2_‐water estimate, for acquisitions with substantially lower SNR this aspect may become an important consideration.

To improve the quality of the separation and fitting itself, further improvements may be possible. Clearly, stimulated echoes and coherences manifest as periodic oscillations are visible in the water signal (e.g. residuals in Figure 2), and these are not taken into account in the mono‐exponential fitting process that discards the first echo. There are strategies available to more fully model the signal behavior based on knowledge of the slice RF profile.^20^, ^36^ This approach could be applied here and may improve the precision of the fitted *T*_2_ (or f.f. via a more precise estimation of the amplitudes *A*_w_, *A*_f_, *c*_w_ and *c*_f_). A seven‐peak model of the fat spectrum was used in the IDEAL separation, with spectral components determined from the literature. There may be opportunity to refine the parameters of the spectral model further by acquiring custom spectra to more precisely define the frequency and relative amplitudes of the spectral components.

## 5 CONCLUSION

Practical and efficient quantitative MRI protocols for tracking muscle pathologies are urgently required. Trials of recently available genetic and stem‐cell‐based experimental therapies for conditions such as Duchenne muscular dystrophy^37^ demand outcome measures with far greater responsiveness to temporal changes than any existing clinical measures of strength or function. In the range of neuromuscular diseases, inflammation, edema, fat and fibrosis are the common mechanisms that can be identified on *ex vivo* pathology and that give rise to the clinical deficit in muscle strength and function. The challenge in this field is to track these changes directly, sensitively, *in vivo* and repeatedly over time to unequivocally determine the statistical efficacy of experimental treatments in cohorts of a realistic size. Finding appropriate ways to use MRI to effectively quantify muscle fibrosis remains an unsolved problem. However, tracking water and fat changes is more immediately tractable and much work has been conducted to date to demonstrate the suitability of chemical‐shift‐based fat quantification in this context.^38^ Methods have also been proposed to measure water changes in the presence of fat, a challenge that IDEAL‐CPMG addresses directly.

In this work the reliability of IDEAL‐CPMG was demonstrated in healthy individuals, and pathological sensitivity examined in a group with HypoPP, a condition where muscle fluid and fat changes are both present. The method was shown to be capable of reproducibly measuring *T*_2_‐water in healthy subjects and identifying *T*_2_ elevations independently of fat in HypoPP patients, with general insensitivity to transmit field variations. In HypoPP, and in other conditions where water‐driven pathologies are important, IDEAL‐CPMG offers an effective acquisition‐based approach to quantifying muscle water *T*_2_ independently of fat. The longitudinal responsiveness of *T*_2_‐water thus obtained as a marker of disease progression, and the comparative time courses of muscle f.f. and *T*_2_‐water changes, are now important avenues of research.
